# Enhancing Rehydrated Rice Husk as Ruminant Feed via Silage Additives: An In Vitro Study

**DOI:** 10.3390/ani16121835

**Published:** 2026-06-14

**Authors:** Chatchai Kaewpila, Julasinee Maensathit, Pairote Patarapreecha, Waroon Khota

**Affiliations:** Faculty of Natural Resources, Rajamangala University of Technology Isan, Sakon Nakhon 47160, Thailand; chatchai.ka@rmuti.ac.th (C.K.); julasinee.ma@rmuti.ac.th (J.M.); pairote.pa@rmuti.ac.th (P.P.)

**Keywords:** rice husk silage, silage additives, rumen fermentation, methane emission intensity, in vitro digestibility

## Abstract

Rice husk is an abundant agricultural by-product with limited use in ruminant feeding due to its high lignocellulosic content and low digestibility. This study compared biological, enzymatic, and chemical additive strategies for improving the ensiling characteristics, chemical composition, and in vitro rumen fermentation of rehydrated rice husk. The experiment was conducted using a completely randomized design with seven treatments: Control (no additive); molasses + *Lacticaseibacillus casei* TH14 (MB); Acremonium cellulase (AC); laccase (LC); AC + LC; AC + LC + MB; and chemical treatment (CM). After 30 days of ensiling, CM reduced fiber contents and increased in vitro dry matter digestibility (IVDMD) and total volatile fatty acid concentrations (*p* < 0.05). Among the biological additives, MB and AC + LC + MB significantly reduced NDF and improved ensiling quality and IVDMD (*p* < 0.05). Notably, AC + LC + MB resulted in the lowest methane emission intensity (44.07 mg/g IVDMD). Although CM, which was included as a chemical-disruption benchmark, was the most effective approach for substrate solubilization, AC + LC + MB improved fermentation characteristics, in vitro digestibility, and methane emission intensity per unit of digested dry matter under the present in vitro conditions.

## Simple Summary

Rice husk is an abundant agricultural by-product, but its high lignocellulosic and silica contents limit its use as ruminant feed. This study evaluated the effects of microbial inoculants, molasses, fibrolytic enzymes, and chemical additives on the fermentation quality and in vitro rumen fermentation of rehydrated rice husk silage. Chemical treatment showed the greatest improvement in fiber degradation and digestibility under the present in vitro conditions. In addition, the combination of molasses, *Lacticaseibacillus casei* TH14, cellulase, and laccase improved silage fermentation, enhanced digestibility, and reduced methane emission intensity per unit of digested dry matter. These findings suggest that additive-treated rice husk silage may have potential as an alternative feed resource for ruminants and may contribute to improved utilization of agricultural by-products. However, further in vivo studies are required to confirm these effects under practical feeding conditions.

## 1. Introduction

Roughage is an indispensable component of the ruminant diet, typically accounting for 60–80% of total intake. It serves as a source of essential nutrients and is crucial for maintaining normal rumen function and health [1]. However, shortages of high-quality roughage during the dry season remain common in tropical regions, particularly in Thailand [2]. Consequently, the utilization of agricultural residues as animal feed has become a vital strategy for sustainable livestock production. Common agricultural by-products used as ruminant feed include rice straw, corn stover, and sugarcane tops. Rice husk, another abundant by-product of the rice milling industry, accounts for approximately 20% of total grain weight [3] and has potential as an alternative roughage source for ruminants [4]. However, its utilization remains limited due to its high lignocellulosic and silica contents, which restrict microbial degradation and nutrient utilization in the rumen [3,5,6].

Ensiling is widely recognized as an effective preservation method for improving the storage stability and feeding value of fibrous agricultural by-products. Successful silage fermentation relies on rapid acidification by lactic acid bacteria (LAB), which suppress undesirable microorganisms and reduce nutrient losses [7]. However, lignocellulosic materials with low water-soluble carbohydrate (WSC) contents, such as rice husk, often exhibit poor fermentation and limited lactic acid production. Therefore, silage additives are commonly applied to enhance fermentation efficiency and improve nutritive value. Among these, LAB inoculants, molasses, and fibrolytic enzymes are used as fermentation stimulants, whereas chemical additives help inhibit undesirable microorganisms and disrupt fiber structures [7,8]. Previous studies have demonstrated that LAB inoculants can improve fermentation characteristics, enhance nutrient preservation, and reduce methane production during ruminal fermentation [9,10]. Xie et al. [11] and Peng et al. [12] reported that the combined application of *Lactiplantibacillus plantarum* and molasses improved the fermentation quality and digestibility of alfalfa silage. Additionally, Li et al. [13] concluded that combining cellulase and laccase improved fermentation quality and modulated the microbial community in mixed corn stover and wet brewer’s grain silage. Chemical pretreatments using alkaline or acidic agents are also effective for disrupting lignin–carbohydrate complexes. For instance, Huang et al. [14] reported that the addition of 0.6% H_2_SO_4_ during ensiling improved nutrient preservation, reduced dry matter (DM) loss, and enhanced the saccharification efficiency of high-moisture corn stover by promoting hemicellulose degradation and increasing WSC yield.

While rehydration is necessary to facilitate the ensiling of dry agricultural residues, the extremely low fermentability and high silica–lignocellulosic complexity of rice husk remain major constraints limiting its utilization as ruminant feed [15,16]. Compared with other crop residues such as rice straw or corn stover, rice husk is considerably more recalcitrant to microbial degradation due to its dense lignified structure and silica-rich outer layer [3]. Although previous studies have demonstrated beneficial effects of LAB, molasses, fibrolytic enzymes, or chemical pretreatments on various fibrous silages [3,11,17], information regarding the direct comparison of biological additive combinations, enzymatic treatments, and chemical additive strategies for improving the fermentation characteristics, nutritive value, and in vitro ruminal fermentation of rehydrated rice husk silage remains scarce. In particular, studies evaluating the combined application of cellulase, laccase, molasses, and LAB inoculants in rice husk silage are very limited.

Therefore, this study compared the effects of molasses combined with *Lacticaseibacillus casei* TH14 (formerly *Lactobacillus casei* TH14), exogenous fibrolytic enzymes, and chemical additives on the ensiling characteristics, chemical composition, and in vitro rumen fermentation of rehydrated rice husk. These additives were hypothesized to improve the nutritive value and in vitro digestibility of rice husk, thereby enhancing its potential as an alternative ruminant feed resource.

## 2. Materials and Methods

### 2.1. Rice Husk Preparation

Ground rice husk samples (passed through a 4 mm screen) were collected from rice mills located in Sakon Nakhon Province, Thailand, in October 2025. Five samples were thoroughly mixed to obtain a homogeneous composite sample, which was then divided into nine portions. The first portion (500 g) was used to analyze the chemical composition and in vitro digestibility prior to ensiling. The second portion (10 g) was used to determine microbial populations and pH before ensiling, whereas the remaining seven portions (1100 g each) were used for silage preparation of the seven experimental treatments.

### 2.2. Design and Silage Preparation

The experiment was conducted using a completely randomized design (CRD), consisting of seven silage additive treatments: Treatment 1: Control (without additives or bacteria); Treatment 2: molasses + *Lacticaseibacillus casei* TH14 (MB); Treatment 3: Acremonium cellulase (AC); Treatment 4: laccase (LC); Treatment 5: AC + LC; Treatment 6: AC + LC + MB; and Treatment 7: chemical treatment (CM). Each treatment was prepared in four independent silos, and each silo served as an individual biological replicate and experimental unit for silage analysis. Prior to ensiling, distilled water (1368 mL) was added to ground rice husk (1100 g) in all treatments to rehydrate the substrate and adjust the dry matter content to approximately 39–40%, which is considered suitable for silage fermentation.

Molasses, used to increase fermentable sugar availability, was a commercial feed-grade product obtained from a local feed supplier in Sakon Nakhon, Thailand, and was applied at 3% on a fresh matter (FM) basis without dilution. The TH14 strain, used as a lactic acid-producing inoculant, was obtained from the culture collection of the Faculty of Natural Resources, Rajamangala University of Technology Isan, Sakon Nakhon Campus, Thailand, and cultured overnight in de Man, Rogosa, and Sharpe (MRS) broth (Difco Laboratories Inc., Detroit, MI, USA) following Pholsen et al. [2]. The inoculum was subsequently suspended in distilled water to obtain a final concentration of 1.0 × 10^8^ CFU per 10 mL. LC and AC were applied to enhance the enzymatic hydrolysis of fibrous substrates. Laccase (1020 LAMU/g; Sigma-Aldrich, St. Louis, MO, USA) was applied directly to the material at 1% on an FM basis without dilution, whereas AC (Meiji Seika Pharma Co., Ltd., Tokyo, Japan), containing glucanase, pectinase, and CMCase activities (7350 U CMCase/g), was dissolved in distilled water (0.2 g in 10 mL) and uniformly sprayed onto 1000 g FM of the prepared material, providing approximately 1.47 U CMCase/g FM substrate. For the CM treatment, NaOH (97% purity; KemAus, New South Wales, Australia) and H_2_SO_4_ (98% purity; KemAus) were separately dissolved in distilled water. The NaOH solution (1% FM basis) was first uniformly sprayed onto the prepared material and thoroughly mixed, after which the treated substrate was allowed to stand for approximately 90 min prior to H_2_SO_4_ application (1.75% FM basis). Subsequently, the H_2_SO_4_ solution was sprayed and thoroughly mixed into the material before ensiling. This stepwise application was intended to allow the alkaline treatment to interact with the lignocellulosic structure prior to acidification. The pH of the chemically treated substrate was approximately 2.0 immediately after the sequential application of NaOH and H_2_SO_4_, indicating that the material remained under strongly acidic conditions prior to ensiling. The CM treatment was included as a chemical reference treatment to provide a benchmark for comparison with the biological additive strategies evaluated in this study. The inclusion levels of fibrolytic enzymes and chemical additives used in the present study were selected based on previous silage studies and adjusted according to the highly recalcitrant nature of rice husk, which is characterized by high lignin and silica contents. Compared with other forage substrates, rice husk generally exhibits lower fermentability and greater resistance to microbial and enzymatic degradation. Therefore, relatively higher application levels of laccase, cellulase, and chemical additives were evaluated in an attempt to enhance lignocellulosic disruption during ensiling. The selected levels were adapted from previous studies involving alfalfa silage, sunn hemp silage, and corn stover silage, with modifications to account for differences in substrate characteristics and structural complexity [17,18,19]. No preliminary dose-optimization experiment was conducted prior to this study. Therefore, the additive inclusion levels evaluated in the present study should be considered exploratory rather than optimized for rice husk silage, and further studies are required to determine the optimal application rates for this substrate.

The treated rice husk was thoroughly mixed with the respective additives, and 500 g portions were packed into laminated nylon–polyethylene bags (Hiryu KN, Asahi Kasei, Tokyo, Japan). The bags were vacuum-sealed using a vacuum packaging machine (SQ-303, Asahi Kasei Pax Corp., Tokyo, Japan) and stored under ambient conditions (25–37 °C). After 30 d of ensiling, all silos were opened for the determination of fermentation quality, chemical composition, microbial populations, and in vitro ruminal digestibility.

### 2.3. Chemical Composition Analysis

Fresh rice husk and silage samples were first oven-dried at 60 °C for 48 h and subsequently milled to pass through a 1 mm screen (MF 10 basic, IKA, Staufen, Germany). Dry matter and ash contents were analyzed following AOAC procedures [20], and organic matter was calculated by difference. Nitrogen concentration was measured using an elemental analyzer (EA3100, Eurovector, Pavia, Italy), and crude protein was estimated as N × 6.25. Fiber fractions, including neutral detergent fiber (NDF) and acid detergent fiber (ADF), were determined with an ANKOM fiber analyzer (ANKOM 200, ANKOM Technology, Macedon, NY, USA) according to the method described by Van Soest et al. [21]. Acid detergent lignin (ADL) was quantified following sulfuric acid digestion [22]. For NDF determination, α-amylase (2500 U/mg; Sigma-Aldrich, St. Louis, MO, USA) and sodium sulfite (98% purity; Kemaus, New South Wales, Australia) were included to facilitate the removal of starch and protein contaminants, respectively.

### 2.4. Measurement of Ensiling Loss, Fermentation End-Product, and Microbial Populations

Dry matter loss was calculated from the difference between the weights recorded at silo sealing and silo opening after 30 d of ensiling and expressed relative to the initial ensiled mass.

For fermentation analyses, aqueous extracts were prepared by blending 10 g of fresh silage with 90 mL of distilled water. The mixtures were maintained at 4 °C for 12 h before being brought to room temperature (25 °C) for pH determination using a pH meter (FiveGo, Mettler-Toledo GmbH, Greifensee, Switzerland). Organic acids, including lactic, acetic, propionic, and butyric acids, were analyzed by gas chromatography (GC-2020, Shimadzu Co., Kyoto, Japan) fitted with a DB-WAX capillary column (30 m × 0.25 mm × 0.25 μm; Agilent Technologies, Santa Clara, CA, USA) and a flame ionization detector, following the procedure described by Kaewpila et al. [23]. Ammonia-N concentration was determined spectrophotometrically using a microplate reader (Infinite 200 PRO, Tecan Trading AG, Grödig, Austria) according to Fawcett and Scott [24].

Microbial populations were quantified in both fresh rice husk and silage samples using standard serial dilution and plating techniques [25]. Briefly, 10 g of sample was mixed with 90 mL of sterile 0.85% NaCl solution and serially diluted to obtain appropriate dilution levels. Aliquots (20 μL) from each dilution were plated onto selective media. LAB were cultured on MRS agar (Difco Laboratories Inc., Detroit, MI, USA) and incubated anaerobically at 30 °C for 48 h. Coliforms and aerobic bacteria were enumerated on blue-light agar (Nissui Seiyaku Ltd., Tokyo, Japan) and nutrient agar (Difco), respectively, after incubation at 30 °C for 72 h. Molds were grown on potato dextrose agar (Nissui Seiyaku Ltd., Tokyo, Japan) and incubated at 30 °C for 3–7 d.

### 2.5. In Vitro Digestibility, Gas Production, and Fermentation Products

The samples were investigated for gas production (GP) and in vitro digestibility of DM (IVDMD), NDF (IVNDFD), and ADF (IVADFD) at 24 h after incubation in triplicate using a gas production technique described by Makkar et al. [26]. The 24 h incubation period was selected to evaluate early-stage ruminal fermentation characteristics and methane production under standardized in vitro conditions for comparative assessment among treatments. Each silage sample obtained from an individual silo was incubated in triplicate to minimize analytical variation. The triplicate incubations were considered technical replicates, and their mean value was used for subsequent statistical analysis. Approximately 0.5 g of each finely ground rice husk or silage sample was weighed into 50 mL serum bottles. To simulate practical feeding conditions in ruminant production systems, where rice husk silage would not typically be fed as the sole dietary component, 0.25 g of concentrate was added equally to all incubation bottles, including the blank bottles, using heat-sealed ANKOM bags (F57). This approach was intended to minimize variation associated with concentrate supply and allow comparisons among treatments to primarily reflect differences in the treated rice husk silages. All bottles were sealed with rubber stoppers and aluminum caps. The concentrate consisted primarily of cassava chips (26.00%), cassava pulp (25.98%), rice bran (19.70%), soybean meal (16.78%), palm kernel meal (10.19%), urea (0.45%), mixed minerals (0.45%), and premix (0.45%), and contained 89.02% DM, 93.42% OM, 14.60% CP, 29.54% NDF, 10.01% ADF, and 3.71% ADL.

Rumen fluid was collected via a stomach tube before the morning feeding from two healthy, mature, male Zebu × Angus (75:25) crossbred beef cattle (mean body weight: 200 ± 15 kg). The cattle were fed a total mixed ration of rice straw and concentrate (70:30 ratio on a DM basis) with free access to clean water. The concentrate contained 12.0% soybean meal, 6.0% rice bran, 7.5% cassava chip, 3.0% molasses, 0.5% urea, 0.5% premix, and 0.5% mixed minerals. The chemical composition of the TMR was 91.59% DM, 92.40% OM, 12.38% CP, 2.18% EE, 52.31% NDF, 31.37% ADF, and 3.75% ADL (DM basis). Following Muizelaar et al. [27], the initial 500 mL of rumen fluid was discarded to minimize saliva contamination. The remaining fluid was immediately transported to the laboratory, pooled, filtered through four layers of cheesecloth, mixed with a buffer solution, and flushed with CO_2_ to maintain anaerobic conditions. The subsequent in vitro incubation was performed as a single experimental run using the pooled rumen fluid.

Each serum bottle received 40 mL of the rumen medium, and the headspace was purged with CO_2_ before incubation in an orbital shaker (SI600, Stuart, Staffordshire, UK) at 39 °C and 60 rpm. The GP was recorded at 2, 4, 8, 16, and 24 h using a calibrated glass syringe, and the gas volumes measured at each interval were cumulatively summed to determine total gas production over the 24 h incubation period. For methane analysis, the gas generated during each interval was continuously transferred into a gas collection bag throughout incubation. At the end of the 24 h incubation period, the pooled gas sample was analyzed by gas chromatography (GC-2020). Intermediate measurements were collected solely for cumulative gas determination, and no gas production kinetics model was fitted. Therefore, treatment comparisons were based on cumulative gas production and methane production over the 24 h incubation period. Methane production was expressed as both mg/g DM and mg/g IVDMD to evaluate total methane production per unit of substrate incubated and methane emission intensity relative to the amount of digested dry matter, respectively. Blank bottles containing rumen medium and concentrate without silage substrate were included during incubation, and gas production values were corrected by subtracting the corresponding blank values. After incubation, the concentrate bags were removed from each bottle prior to digestibility determination. The remaining rice husk silage residues were then filtered using F57 bags, dried at 100 °C for 24 h, and weighed to calculate IVDMD. These bags were then sequentially extracted with NDF and ADF solutions to determine IVNDFD and IVADFD, respectively. Volatile fatty acid (VFA) concentrations were analyzed using gas chromatography, following the procedure of Kaewpila et al. [23].

### 2.6. Statistical Analysis

Prior to statistical analysis, the assumptions of normality and homogeneity of variance were evaluated using the Shapiro–Wilk and Levene’s tests, respectively. The data obtained from silages were analyzed using an ANOVA procedure of SAS Version 6.12 (SAS Institute Inc., Cary, NC, USA). The statistical model is as follows:

Y_ij_ = µ + T_i_ + ɛ_ij_

where Y_ij_ is the observation for treatment i and replicate j, μ is the overall mean, T_i_ is the additive effect (i = 1–7), and ɛ_ij_ is the residual error. Mean differences were compared using Duncan’s New Multiple Range Test at a significance level of *p* < 0.05 [28].

## 3. Results

### 3.1. Chemical Composition and Microbial Populations of Raw Rice Husk Before Ensiling

The chemical composition and microbial counts of rice husk prior to ensiling are presented in Table 1. The rice husk contained 89.63% DM, 3.14% CP, and a high fiber content, with NDF, ADF, and ADL at 88.13%, 64.72%, and 28.18% on a DM basis, respectively. Regarding the epiphytic microbial populations, the counts of LAB, aerobic bacteria, and coliforms were 1.0 × 10^4^, 8.0 × 10^3^, and 8.8 × 10^4^ CFU/g FM, respectively, while molds were not detected. The initial pH of the rice husk was 5.8. The in vitro digestibility, including IVDMD, IVNDFD, and IVADFD, was 94.15, 46.97, and 77.02 g/kg DM, respectively. The total gas production was 24.25 mL/g DM, and methane emissions in the units of mg/g DM and mg/g IVDMD were 7.92 and 83.59, respectively. In addition, acetic acid, propionic acid, butyric acid, and valeric acid concentrations were 21.54, 17.42, 6.83, and 0.77 mmol/L, respectively.

### 3.2. Chemical Composition of Rehydrated Rice Husk Silages

There were no significant differences (*p* > 0.05) observed in DM and CP contents across all treatments (Table 2). However, the OM content was significantly affected by the treatments (*p* < 0.001), with the MB, AC + LC, and AC + LC + MB groups exhibiting the highest OM values compared to the control. Compared with the control, the CM treatment reduced NDF, ADF, and ADL concentrations by approximately 19.1%, 13.0%, and 13.6%, respectively. Among the biological additive treatments, the MB and AC + LC + MB groups also exhibited lower NDF concentrations than the control treatment, representing reductions of approximately 6.5% and 6.3%, respectively. Furthermore, the AC + LC + MB treatment resulted in a significantly lower ADF content (60.69%) compared with the LC group (64.08%; *p* < 0.001). For ADL, the CM treatment was significantly more effective than the other treatments in reducing lignin content (*p* = 0.032).

### 3.3. Dry Matter Loss and Fermentation End-Products of Rehydrated Rice Husk Silages

Significant differences were observed among treatments for all fermentation parameters (*p* < 0.001) (Table 3). Overall, treatments containing molasses and LAB (MB and AC + LC + MB), as well as the CM treatment, showed superior fermentation quality compared with the control and enzyme-only treatments. The MB, AC + LC + MB, and CM treatments resulted in the lowest DM losses (4.48–7.65 g/kg), whereas AC + LC exhibited the greatest loss (28.41 g/kg). Compared with the control treatment, DM loss was reduced by approximately 67.4%, 63.4%, and 78.6% in the MB, AC + LC + MB, and CM treatments, respectively, whereas the AC + LC treatment increased DM loss by approximately 35.9%. Similarly, these treatments also produced lower pH values, indicating more efficient acidification during ensiling. Notably, the CM treatment exhibited an extremely low pH of 2.00, which was substantially lower than those observed in all biological additive treatments (3.75–4.23), together with low concentrations of butyric acid and ammonia-N, indicating the strongest acidification response among all treatments. Among the biological additive treatments, MB showed the greatest improvement in fermentation characteristics, as evidenced by the highest concentrations of lactic acid, acetic acid, and propionic acid. In contrast, the control, LC, and AC + LC treatments exhibited higher butyric acid and ammonia-N concentrations, suggesting less desirable fermentation. Furthermore, MB and AC + LC + MB produced low pH values (<4.0) together with minimal butyric acid and ammonia-N concentrations, indicating improved fermentation quality compared with the other treatments.

### 3.4. Microbial Populations of Rehydrated Rice Husk Silages

The biological additive treatments exhibited significantly higher (*p* < 0.05) LAB populations than the control, with AC + LC showing the highest LAB count among all treatments (6.77 log10 CFU/g FM), representing an increase of 1.45 log units compared with the control treatment (5.32 log10 CFU/g FM) (Table 4). In contrast, the control treatment had significantly higher (*p* < 0.01) aerobic bacteria counts compared to all additive-treated silages. Notably, no LAB or aerobic bacteria were detected in the CM treatment, making it the only treatment in which both microbial groups were completely undetectable after 30 days of ensiling. Additionally, coliform bacteria and molds were not detected (ND) in any of the treatments.

### 3.5. In Vitro Ruminal Degradation and Gas Production of Rehydrated Rice Husk Silages

The in vitro fermentation parameters of rice husk silages after 24 h of incubation are presented in Table 5. Significant differences were observed among treatments for all measured parameters (*p* < 0.05). Overall, the CM treatment exhibited the highest IVDMD (239.12 g/kg DM) and gas production (56.81 mL/g DM). Compared with the control treatment, IVDMD, IVNDFD, and IVADFD increased by approximately 198.3%, 21.8%, and 12.1%, respectively, indicating that the effect of CM was substantially greater for overall dry matter digestibility than for fiber digestibility parameters. The LC treatment exhibited the highest IVNDFD (81.04 g/kg DM), whereas the MB treatment showed the highest IVADFD (97.36 g/kg DM). These responses differed from the ranking observed for IVDMD, in which the CM treatment showed the highest value, indicating that treatment effects varied depending on the digestibility parameter and fiber fraction evaluated. Methane production expressed as mg/g DM was highest in the CM treatment, whereas AC, AC + LC, and AC + LC + MB produced lower methane values. However, treatment ranking differed when methane emission was expressed relative to digested dry matter (mg/g IVDMD). Although the CM treatment produced the highest methane production on a DM basis, the AC + LC + MB treatment exhibited the lowest methane emission intensity (44.07 mg/g IVDMD), while the LC treatment showed the highest value (95.61 mg/g IVDMD). These results indicate that methane production expressed per unit of substrate and per unit of digested dry matter may provide different perspectives on treatment responses because digestibility varied among treatments.

### 3.6. Volatile Fatty Acid Profiles of Rehydrated Rice Husk Silages

The VFA profiles of rehydrated rice husk silages after 24 h of incubation with rumen inoculum are shown in Table 6. Significant differences among treatments were observed for total VFA (TVFA) concentration, butyric acid, and valeric acid proportions (*p* < 0.05). Overall, the LC, CM, and MB treatments resulted in greater TVFA concentrations than the control and other additive treatments, indicating enhanced ruminal fermentation. Among these, the LC treatment produced the highest TVFA concentration (63.58 mmol/L), followed by CM and MB. Compared with the control treatment, TVFA concentration increased by approximately 30.8%, 26.0%, and 20.6% in the LC, CM, and MB treatments, respectively. The molar proportions of acetic acid and propionic acid were not significantly affected by treatment (*p* > 0.05). Notably, although total VFA concentrations differed significantly among treatments, the molar proportions of acetic acid and propionic acid were unaffected. In contrast, the proportions of butyric acid and valeric acid varied among treatments. The control, MB, and AC treatments exhibited relatively higher butyric acid proportions, whereas AC + LC + MB and CM showed lower values. Compared with the control treatment, the butyric acid proportion decreased by approximately 22.7% and 21.6% in the AC + LC + MB and CM treatments, respectively. In addition, the CM treatment resulted in the lowest valeric acid proportion, representing an approximately 55.0% reduction compared with the control treatment.

## 4. Discussion

### 4.1. Chemical Composition and Microbial Populations of Rice Husk Before Ensiling

Rice husk is one of the most abundant agricultural by-products in rice-producing regions, offering significant potential as a low-cost, alternative roughage source for ruminant production. However, its utilization is limited by a poor nutritional profile. As observed in our initial analysis, the raw rice husk contained a low CP content (3.14%), high structural carbohydrates (88.13% NDF, 64.72% ADF and 28.18% ADL), and low IVDMD (94.15 g/kg DM) (Table 1). These findings are consistent with previous reports. Rosani et al. [5] reported that raw rice husk contains 3.8% CP and 19.57% lignin, while Friedman [15] found that rice husk contains 2.33% CP, 79.70% NDF, 67.74% ADF, and 15.39% ADL. In contrast, Begna et al. [29] reported a higher CP (6.8%) and lower ADL (14.4%) compared to our findings. The variation in the chemical composition of rice husk might be attributed to differences in rice variety, environmental conditions, maturity at harvest, and post-harvest processing [30].

### 4.2. Chemical Composition of Rice Husk Silage

Organic matter content was influenced not only by additive composition but also by the preservation and loss of organic constituents during ensiling. Although the CM treatment received inorganic additives, its OM content was only slightly lower than that of the control treatment. In contrast, the higher OM contents observed in the MB and AC + LC + MB treatments may be associated with their lower dry matter losses and improved preservation of organic matter during fermentation. Regarding fiber fractions, the CM treatment exhibited the greatest reduction in NDF, ADF, and ADL contents among all treatments (*p* < 0.05) (Table 2). The sequential application of NaOH followed by H_2_SO_4_ may have altered the physicochemical characteristics of the lignocellulosic matrix, thereby increasing substrate accessibility and partially solubilizing structural carbohydrates. Alkaline treatment has previously been reported to disrupt ester linkages and loosen lignocellulosic structures, whereas acidic conditions may further contribute to hemicellulose solubilization and structural modification [31,32]. However, partial neutralization between residual NaOH and H_2_SO_4_ may also have occurred during the process, potentially resulting in the formation of inorganic salts such as Na_2_SO_4_. Therefore, the observed effects in the CM treatment were likely associated with combined chemical modification rather than solely distinct alkaline or acid mechanisms. However, these proposed mechanisms were inferred from previous studies, as structural and physicochemical changes in the lignocellulosic matrix were not directly evaluated in the present study. The extremely low pH (approximately 2.0) observed immediately after treatment further suggests that residual chemical effects may have contributed to the subsequent fermentation responses.

Compared with the control, the MB and AC + LC + MB treatments exhibited the lowest NDF content. This reduction is likely associated with the preservation of soluble components in additive-treated silages, which lowered the relative proportion of structural carbohydrates [33]. In molasses-containing treatments, readily fermentable sugars stimulated rapid proliferation of LAB, resulting in increased lactic acid production and a faster decline in pH. The acidic conditions promoted hemicellulose hydrolysis and partial solubilization of cell wall components, contributing to NDF reduction [12,33]. The combined treatment (AC + LC + MB) may have resulted in greater reductions in fiber fractions due to potential complementary effects among fibrolytic enzymes, molasses, and LAB. Fibrolytic enzymes may have contributed to increased substrate accessibility, whereas organic acids produced during LAB fermentation may have further supported the degradation of structural carbohydrates [34]. However, enzyme activity, microbial interactions, and structural changes in the lignocellulosic matrix were not directly evaluated in the present study. These findings agree with previous studies reporting that the combined use of LAB, fermentable carbohydrates, and fibrolytic enzymes results in greater NDF reduction than the control [12,34,35].

However, CP content remained stable across treatments (*p* > 0.05), indicating that the fermentation process effectively preserved the primary nutrients of the rehydrated rice husk. This observation is consistent with previous studies [11,12]. In contrast, other studies have shown increases in CP content when molasses and LAB were applied [36].

### 4.3. Dry Matter Loss and Fermentation End-Products of Rice Husk Silage

Overall, additive treatments markedly improved the fermentation characteristics and dry matter preservation of rice husk silage compared with the control treatment (*p* < 0.001) (Table 3). In particular, treatments containing molasses and LAB exhibited lower DM losses and improved acidification during ensiling, indicating more efficient fermentation and nutrient preservation. This improvement was likely associated with the availability of readily fermentable carbohydrates supplied by molasses, which stimulated rapid LAB proliferation and enhanced organic acid production, thereby limiting nutrient losses caused by undesirable microbial activity [8,12].

Rapid acidification is essential for suppressing clostridial activity and proteolysis during ensiling [37]. In the present study, the CM treatment exhibited the lowest pH, likely due to the strong acidification induced by H_2_SO_4_ application. Similarly, the MB and AC + LC + MB treatments also showed low pH values (<4.0), suggesting improved fermentation efficiency. The enhanced acidification observed in these treatments may have been associated with the combined effects of molasses, LAB, and fibrolytic enzymes. Molasses provided fermentable substrates for LAB growth, while cellulase and laccase may have partially degraded structural carbohydrates and increased the availability of soluble sugars for microbial fermentation. Consequently, LAB rapidly converted available sugars into organic acids, particularly lactic acid, resulting in accelerated pH reduction [38]. In contrast, the control and enzyme-only treatments exhibited relatively higher pH values, indicating less efficient fermentation. This may be attributed to the inherent characteristics of rice husk, which is highly lignified and contains substantial silica, thereby restricting microbial degradation and limiting fermentation efficiency [15,16,30].

Regarding fermentation end-products, MB treatments showed greater organic acid production, particularly lactic, acetic, and propionic acids, indicating enhanced LAB fermentation activity and improved silage preservation [7]. Conversely, the control, LC, and AC + LC treatments exhibited higher butyric acid concentrations and ammonia-N levels, suggesting undesirable clostridial fermentation and increased protein degradation. Clostridia can proliferate under conditions of insufficient acidification and subsequently metabolize sugars and lactic acid into butyric acid while promoting proteolysis [14,39]. Elevated butyric acid concentrations therefore indicate a shift away from desirable lactic acid fermentation toward less efficient secondary fermentation pathways [7]. In contrast, the MB, AC + LC + MB, and CM treatments exhibited lower butyric acid and ammonia-N concentrations, reflecting improved fermentation efficiency and reduced nutrient degradation during ensiling. Although lactic acid and acetic acid were detected in the CM treatment, the mechanisms responsible for their occurrence could not be clearly determined from the present data. Given the extremely low pH of the chemically treated material and the absence of detectable viable microorganisms at silo opening, the origin of these organic acids remains uncertain. Consequently, the organic acid concentrations detected in the CM treatment should not be interpreted as direct evidence of microbial silage fermentation. Among the biological additive treatments, MB and AC + LC + MB showed the most favorable fermentation profiles, characterized by low pH together with minimal butyric acid and ammonia-N concentrations. These findings suggest that combining molasses, LAB, and fibrolytic enzymes may effectively enhance the preservation and fermentation quality of rice husk silage.

Collectively, the present findings indicate that molasses-containing additive strategies, particularly when combined with LAB and fibrolytic enzymes, can improve fermentation quality and reduce nutrient losses during rice husk ensiling. These results are consistent with previous studies reporting superior fermentation characteristics in silages treated with combined additives compared with single or untreated silages [12,19].

### 4.4. Microbial Populations of Rice Husk Silage

The microbial populations observed in this study clearly demonstrate that additive treatments markedly influenced the fermentation ecology of rice husk silage (Table 4). The higher LAB populations in biological additive treatments indicate that these additives promoted the establishment of a dominant LAB community, which is essential for efficient silage fermentation. In particular, molasses-containing treatments likely provided readily fermentable carbohydrates, thereby enhancing LAB proliferation and accelerating lactic acid production [37,40].

The increased LAB counts in enzyme-treated silages can be explained by the action of fibrolytic enzymes, which partially degrade structural carbohydrates and release soluble sugars that serve as substrates for LAB growth [13]. The relatively higher LAB population observed in the combined enzyme treatment (AC + LC) may indicate complementary effects of cellulase and laccase in enhancing fiber disruption and improving substrate accessibility for microbial fermentation.

In contrast, the significantly higher aerobic bacteria counts in the control treatment indicate less efficient anaerobic fermentation and delayed acidification. Under such conditions, oxygen-tolerant microorganisms can persist and compete with LAB for available substrates, thereby reducing fermentation efficiency [7]. The lower LAB population observed in the control treatment further supports the limited fermentation activity in the absence of additives.

The absence of detectable LAB and aerobic bacteria in the CM treatment suggests that chemical additives (NaOH combined with H_2_SO_4_) created conditions unfavorable for microbial survival. Ensiling with acids or alkalis induces rapid and strong chemical acidification, which directly lowers pH, thereby inhibiting both beneficial and undesirable microorganisms, while simultaneously disrupting lignocellulosic linkages and enhancing biomass usability [33]. In addition, partial neutralization between residual NaOH and H_2_SO_4_ may have generated inorganic salts, such as Na_2_SO_4_, which together with the extremely acidic conditions likely contributed to the absence of detectable microbial populations in the CM treatment. Furthermore, the absence of coliform bacteria and molds across all treatments indicates that the ensiling conditions were generally effective in suppressing spoilage microorganisms. However, the present study did not include microbial community analysis, strain-specific quantification, or molecular tracking techniques to confirm the persistence and specific activity of *Lc. casei* TH14 throughout the ensiling process. Therefore, the observed improvements could not be directly attributed solely to *Lc. casei* TH14 and were more likely associated with combined interactions among molasses, LAB inoculation, fibrolytic enzymes, and fermentation dynamics. Further studies involving microbial profiling and strain-specific analyses are required to clarify the specific role of *Lc. casei* TH14 during ensiling and ruminal fermentation.

### 4.5. In Vitro Ruminal Degradation and Gas Production of Rehydrated Rice Husk Silage

Overall, additive treatments influenced the in vitro digestibility and rumen fermentation characteristics of rehydrated rice husk silage (Table 5). Among all treatments, the CM treatment exhibited the greatest improvement in digestibility and gas production, indicating enhanced disruption of the lignocellulosic structure and increased substrate availability for rumen microorganisms. The sequential application of NaOH and H_2_SO_4_ likely promoted alkaline swelling and acid hydrolysis of lignocellulosic linkages, thereby enhancing the solubilization of structural carbohydrates. Previous studies have similarly reported that chemical pretreatment can disrupt lignin–carbohydrate complexes and improve the digestibility of fibrous residues [17]. Ghebriel et al. [41] also reported increased IVDMD in sorghum silage treated with NaOH.

However, the CM treatment in the present study was primarily intended to serve as a positive benchmark for maximal fiber disruption rather than as a directly applicable feeding strategy under practical farm conditions. Although chemical pretreatment substantially improved digestibility, its large-scale application may be constrained by handling safety, corrosiveness, chemical residue concerns, equipment requirements, and environmental management considerations. Moreover, the extremely low pH of the CM-treated substrate (pH = 2.0) was evaluated only under buffered in vitro rumen conditions (pH ≈ 7.0). Therefore, further studies are required to determine whether pH adjustment or additional neutralization procedures would be necessary before practical in vivo application. In addition, in vivo application of the CM treatment would require careful evaluation of chemical residues, animal safety, and dietary buffering considerations prior to practical use. From a practical perspective, the biological additive combinations evaluated in this study, particularly AC + LC + MB, represent a more feasible strategy for improving rice husk utilization under the present experimental conditions.

Among the biological additive treatments, molasses-containing silages generally showed improved IVDMD compared with the control treatment, suggesting beneficial effects of combining fermentable carbohydrates, LAB activity, and fibrolytic enzymes during ensiling. Previous studies have also demonstrated improved digestibility in silages treated with molasses, LAB, and fibrolytic enzymes [35,36]. The improved digestibility observed in the present study may be associated with partial degradation of lignocellulosic structures and increased availability of soluble carbohydrates, which could subsequently facilitate microbial colonization and substrate utilization during ruminal fermentation [42]. In contrast, the control AC, and AC + LC treatments exhibited lower digestibility values, which may reflect the highly recalcitrant nature of rice husk. The high lignin and silica contents of rice husk likely restrict microbial attachment and enzymatic hydrolysis, thereby limiting degradation efficiency [15]. These treatments also retained relatively higher fiber fractions, which may have further contributed to reduced digestibility. Although additive treatments improved digestibility relative to the control, the overall IVDMD values remained relatively low from a nutritional perspective. This limitation likely reflects the highly recalcitrant nature of rice husk, which contains substantial lignin and silica fractions that restrict microbial degradation even after additive application.

Interestingly, the LC treatment showed the highest IVNDFD. This may be attributed to the enzymatic degradation of lignocellulose, which has been reported to modify the structural linkages between cellulose and hemicellulose. Such modifications may increase substrate accessibility to rumen microorganisms and thereby contribute to improved NDF digestibility [43]. In contrast, the MB treatment resulted in the highest IVADFD, which may be associated with the presence of neutral detergent solubles, including WSC in molasses, that are readily utilized by rumen microorganisms [36]. However, microbial attachment, colonization, and structural accessibility mechanisms were not directly evaluated in the present study. These differences in fiber degradability among treatments were subsequently reflected in the ruminal fermentation kinetics and gas production profiles.

It should also be noted that digestibility in the present study were evaluated after 24 h of incubation, which may underestimate the degradation potential of highly lignified substrates such as rice husk. Longer incubation periods (48–72 h) are commonly used to evaluate slowly degradable fiber fractions and could potentially result in greater digestibility estimates. Therefore, future studies involving extended incubation periods are warranted to further investigate the long-term degradation kinetics and fermentation characteristics of treated rice husk silage.

Gas production serves as an indirect indicator of ruminal fermentation kinetics and microbial activity during substrate degradation [44]. In the present study, the higher gas production observed in the CM treatment was consistent with its improved digestibility and enhanced substrate fermentability. Similar increases in gas production following chemical pretreatment have been reported previously [45].

While the CM treatment produced the highest methane production when expressed as mg/g DM, methane production should also be evaluated relative to digestibility. When expressed as mg/g IVDMD, the AC + LC + MB treatment exhibited the lowest methane emission intensity (44.07 mg/g IVDMD), indicating lower methane production per unit of digested substrate. However, treatment rankings differed depending on the methane expression unit. The lower methane emission intensity observed in the AC + LC + MB treatment may be associated with the combined application of fibrolytic enzymes, molasses, and LAB during the ensiling process. Previous studies have suggested that improvements in substrate utilization and fermentation efficiency may influence methane production during ruminal fermentation [3,46]. In addition, the reduced methane emission observed in the AC + LC + MB treatment may be associated with possible changes in ruminal fermentation patterns and microbial activity during substrate degradation. However, the present study did not include microbial community analysis or methanogen quantification; therefore, the underlying microbial mechanisms responsible for methane mitigation could not be directly confirmed. Further studies involving microbial profiling and methanogen characterization are required to clarify the specific microbial interactions associated with these additive strategies. Overall, these findings suggest that the combined application of AC + LC + MB not only improves rice husk digestibility but also enhances fermentation efficiency by lowering methane emission intensity under in vitro conditions, highlighting its potential for improving rice husk utilization as a ruminant feed resource.

### 4.6. Volatile Fatty Acid Concentrations of Rice Husk Silage

Volatile fatty acids are the primary end-products of ruminal carbohydrate fermentation, produced through the microbial degradation of structural and non-structural carbohydrates into short-chain fatty acids, CO_2_, and H_2_. The VFA profile in the rumen is a key indicator of microbial metabolic activity and the energy supply available to the ruminant [47]. Among the treatments, LC, CM, and MB exhibited higher total VFA concentrations than the control and other additive treatments, suggesting enhanced substrate fermentability and microbial activity during ruminal fermentation (Table 6). In particular, the elevated TVFA concentration observed in the CM treatment may be associated with improved substrate accessibility resulting from chemical disruption of lignocellulosic structures. Similarly, the increased TVFA concentrations in molasses-containing and enzyme-treated silages may reflect enhanced availability of fermentable substrates for rumen microorganisms.

The relatively unchanged proportions of acetic and propionic acids across treatments indicate that the additives had limited effects on the major ruminal fermentation pathways. In contrast, the significantly reduced proportions of butyric and valeric acids in the CM and AC + LC + MB treatments may reflect differences in fermentation patterns among treatments. However, the underlying mechanisms responsible for these responses were not directly evaluated in the present study.

Several limitations of the present study should be acknowledged. Although the CM treatment markedly improved digestibility, its practical application under farm conditions may be limited due to handling safety, corrosiveness, and potential chemical residue concerns. Therefore, the biological additive combinations evaluated in this study may represent a more practical approach for improving rice husk utilization.

The present study demonstrated that additive treatments improved the fermentation characteristics, digestibility, and methane emission intensity of rehydrated rice husk silage under in vitro conditions. However, the underlying microbial mechanisms were not directly evaluated, and the findings were obtained using an in vitro rumen fermentation system, which may not fully reflect in vivo ruminal dynamics, animal responses, or long-term feeding performance. Furthermore, despite the observed improvements in fermentation characteristics and digestibility, the overall feeding value of rice husk remained limited due to its inherently high lignocellulosic and silica contents.

Additional limitations should also be considered. The in vitro incubation was conducted as a single experimental run using pooled rumen fluid from two donor cattle; therefore, donor-animal variation and incubation-run variation were not incorporated into the statistical model. Moreover, several parameters relevant to the interpretation of rice husk utilization and silage quality, including silica or acid-insoluble ash, WSC, buffering capacity, aerobic stability, and mineral composition (e.g., Na and S), were not determined in the present study. These measurements could provide additional insight into substrate characteristics, fermentation responses, and practical feeding considerations. Future studies should include independent incubation runs, additional donor animals, and a broader range of physicochemical measurements to further validate and expand upon the present findings.

## 5. Conclusions

Based on the results, additive application improved the fermentation characteristics and in vitro digestibility of rehydrated rice husk silage, although the overall feeding value of rice husk remained limited due to its highly recalcitrant lignocellulosic structure. Chemical treatment was the most effective approach for enhancing acidification and improving IVDMD (up to 239 g/kg). However, the combined application of molasses and *Lc. casei* TH14, either alone or in combination with cellulase and laccase, improved ensiling quality and in vitro digestibility while reducing fiber fractions. In particular, the combination of molasses, *Lc. casei* TH14, cellulase, and laccase was associated with the lowest methane emission intensity when methane production was expressed as mg/g IVDMD under the present in vitro conditions. Further in vivo studies are required to evaluate the practical applicability of these additive strategies and to improve the utilization efficiency of rice husk in ruminant feeding systems.

## Figures and Tables

**Table 1:** Chemical composition, pH, microbial count, and in vitro rumen parameters at 24 h incubation of rice husk before ensiling.

Item		Rice Husk
Chemical composition (% on DM)	DM, %	89.63
	OM	95.28
	CP	3.14
	NDF	88.13
	ADF	64.72
	ADL	28.18
pH		5.80
Microbial counts (CFU/g FM)	Lactic acid bacteria	1.00 × 10^4^
	Aerobic bacteria	8.00 × 10^3^
	Coliform	8.80 × 10^4^
	Mold	ND
In vitro parameters	IVDMD, g/kg DM	94.15
	IVNDFD, g/kg DM	46.97
	IVADFD, g/kg DM	77.02
	Total gas production (mL/g DM)	24.25
	Methane production (mg/g DM)	7.92
	Methane production (mg/g IVDMD)	83.59
	Acetic acid (mmol/L)	21.54
	Propionic acid (mmol/L)	17.42
	Butyric acid (mmol/L)	6.83
	valeric acid (mmol/L)	0.77

DM, dry matter; OM, organic matter; CP, crude protein; NDF, neutral detergent fiber; ADF, acid detergent fiber; ADL, acid detergent lignin; CFU, colony forming unit; ND, not detected; FM, fresh matter; IVDMD, in vitro dry matter digestibility; IVNDFD, in vitro neutral detergent fiber digestibility; IVADFD, in vitro acid detergent fiber digestibility.

**Table 2:** Chemical composition of rehydrated rice husk silage after 30 days of ensiling.

Treatments	DM	OM	CP	NDF	ADF	ADL
	%	% on DM				
Control	40.81 ± 0.85	86.18 ± 0.26 ^c^	3.93 ± 0.05	89.32 ± 1.22 ^a^	62.68 ± 0.79 ^ab^	26.94 ± 1.16 ^a^
MB	38.73 ± 0.85	90.08 ± 0.36 ^a^	3.46 ± 0.43	83.52 ± 0.67 ^b^	61.31 ± 0.44 ^ab^	25.64 ± 0.75 ^ab^
AC	38.33 ± 0.83	88.14 ± 0.19 ^b^	3.44 ± 0.08	90.91 ± 0.27 ^a^	63.61 ± 1.38 ^ab^	26.98 ± 1.18 ^a^
LC	40.20 ± 1.39	88.65 ± 0.37 ^ab^	3.86 ± 0.55	90.22 ± 2.10 ^a^	64.08 ± 3.66 ^a^	27.08 ± 3.34 ^a^
AC + LC	39.75 ± 1.48	89.91 ± 2.11 ^a^	3.72 ± 0.05	90.99 ± 2.47 ^a^	63.59 ± 1.32 ^ab^	27.30 ± 1.19 ^a^
AC + LC + MB	40.60 ± 1.81	89.91 ± 0.83 ^a^	3.84 ± 0.32	83.73 ± 1.07 ^b^	60.69 ± 1.21 ^b^	25.70 ± 1.42 ^ab^
CM	39.33 ± 2.49	85.96 ± 0.38 ^c^	3.65 ± 0.45	72.24 ± 1.49 ^c^	54.53 ± 1.59 ^c^	23.28 ± 1.04 ^b^
SEM	0.750	0.450	0.169	0.753	0.895	0.828
*p*-value	0.210	<0.001	0.292	<0.001	<0.001	0.032

DM, dry matter; OM, organic matter; CP, crude protein; NDF, neutral detergent fiber; ADF, acid detergent fiber; ADL, acid detergent lignin; MB, molasses + *Lc. casei* TH14; AC, Acremonium cellulase; LC, laccase; AC + LC, Acremonium cellulase + laccase; AC + LC + MB, Acremonium cellulase + laccase+ molasses + *Lc. casei* TH14; CM, chemical; SEM, standard error of the means. Data are presented as mean ± standard deviation. Values are means of four silage samples. ^a–c^ Means within columns with different superscripts differ at *p* < 0.05.

**Table 3:** Dry matter losses and fermentation end-products of rehydrated rice husk silage after 30 days of ensiling.

Treatments	DM Loss	pH	Lactic Acid	Acetic Acid	Propionic Acid	Butyric Acid	Ammonia-N
	g/kg		g/kg DM				g/kg DM
Control	20.91 ± 1.23 ^ab^	4.46 ± 0.03 ^a^	12.66 ± 7.42 ^d^	17.30 ± 6.99 ^b^	5.02 ± 0.51 ^b^	17.26 ± 8.04 ^a^	0.25 ± 0.06 ^a^
MB	6.81 ± 0.15 ^c^	3.78 ± 0.02 ^c^	38.32 ± 2.34 ^a^	25.24 ± 1.52 ^a^	6.58 ± 0.12 ^a^	2.77 ± 0.06 ^c^	0.02 ± 0.01 ^e^
AC	23.66 ± 1.23 ^ab^	4.04 ± 0.02 ^b^	17.50 ± 1.80 ^cd^	15.99 ± 0.80 ^b^	4.84 ± 0.13 ^b^	11.98 ± 0.78 ^b^	0.14 ± 0.03 ^bc^
LC	15.72 ± 3.06 ^bc^	4.15 ± 0.23 ^b^	20.00 ± 2.69 ^c^	15.51 ± 1.57 ^b^	5.32 ± 0.63 ^b^	17.87 ± 7.16 ^a^	0.10 ± 0.01 ^cd^
AC + LC	28.41 ± 2.85 ^a^	4.23 ± 0.24 ^b^	27.21 ± 8.60 ^b^	14.69 ± 0.88 ^b^	5.20 ± 0.22 ^b^	18.15 ± 4.04 ^a^	0.18 ± 0.07 ^b^
AC + LC + MB	7.65 ± 0.51 ^c^	3.75 ± 0.05 ^c^	23.38 ± 1.07 ^bc^	13.68 ± 0.37 ^b^	5.09 ± 0.58 ^b^	1.96 ± 0.08 ^c^	0.03 ± 0.02 ^e^
CM	4.48 ± 0.30 ^c^	2.00 ± 0.00 ^d^	2.93 ± 0.26 ^e^	22.50 ± 1.74 ^a^	4.71 ± 0.30 ^b^	2.49 ± 0.34 ^c^	0.07 ± 0.02 ^de^
SEM	3.789	0.065	2.285	1.442	0.204	1.683	0.019
*p*-value	0.001	<0.001	<0.001	<0.001	<0.001	<0.001	<0.001

DM, dry matter; Ammonia-N, ammonia nitrogen; MB, molasses + *Lc. casei* TH14; AC, Acremonium cellulase; LC, laccase; AC + LC, Acremonium cellulase + laccase; AC + LC + MB, Acremonium cellulase + laccase+ molasses + *Lc. casei* TH14; CM, chemical; SEM, standard error of the means. Data are presented as mean ± standard deviation. Values are means of four silage samples. ^a–e^ Means within columns with different superscripts differ at *p* < 0.05.

**Table 4:** Microbial populations of rehydrated rice husk silage after 30 days of ensiling.

Treatments	LAB	Aerobic Bacteria	Coliform Bacteria	Molds
	log10 CFU/g FM			
Control	5.32 ± 0.98 ^b^	4.78 ± 0.07 ^a^	ND	ND
MB	6.06 ± 0.09 ^ab^	3.98 ± 0.05 ^b^	ND	ND
AC	6.26 ± 1.02 ^ab^	4.01 ± 0.08 ^b^	ND	ND
LC	5.63 ± 0.99 ^ab^	3.72 ± 0.10 ^c^	ND	ND
AC + LC	6.77 ± 0.13 ^a^	4.02 ± 0.12 ^b^	ND	ND
AC + LC + MB	6.39 ± 0.27 ^ab^	4.07 ± 0.31 ^b^	ND	ND
CM	ND ^c^	ND ^d^	ND	ND
SEM	0.352	0.068	-	-
*p*-value	<0.001	<0.001	-	-

LAB, lactic acid bacteria; CFU, colony forming unit; FM, fresh matter; MB, molasses + *Lc. casei* TH14; AC, Acremonium cellulase; LC, laccase; AC + LC, Acremonium cellulase + laccase; AC + LC + MB, Acremonium cellulase + laccase+ molasses + *Lc. casei* TH14; CM, chemical; ND, no colonies detected on the agar plates at any dilution level examined; SEM, standard error of the means. Data are presented as mean ± standard deviation. Values are means of four silage samples. ^a–c^ Means within columns with different superscripts differ at *p* < 0.05.

**Table 5:** In vitro ruminal degradation, gas production, and methane production of rehydrated rice husk silages after 24 h of incubation with rumen inoculum.

Treatments	IVDMD	IVNDFD	IVADFD	GP	Methane Production	
	g/kg DM			mL/g DM	mg/g DM	mg/g IVDMD
Control	80.16 ± 9.77 ^c^	54.92 ± 11.13 ^b^	40.53 ± 8.82 ^c^	33.91 ± 3.33 ^b^	6.40 ± 0.55 ^cd^	80.65 ± 13.09 ^ab^
MB	144.54 ± 12.14 ^b^	62.07 ± 14.93 ^ab^	97.36 ± 6.91 ^a^	33.23 ± 1.81 ^b^	10.28 ± 1.02 ^b^	71.12 ± 3.70 ^abc^
AC	77.20 ± 1.22 ^c^	46.68 ± 15.49 ^b^	51.42 ± 2.79 ^bc^	25.38 ± 3.05 ^b^	5.56 ± 1.81 ^d^	71.73 ± 22.32 ^abc^
LC	96.13 ± 3.16 ^c^	81.04 ± 20.60 ^a^	84.93 ± 40.92 ^ab^	32.20 ± 5.40 ^b^	9.22 ± 2.37 ^bc^	95.61 ± 22.98 ^a^
AC + LC	75.37 ± 33.63 ^c^	46.88 ± 24.87 ^b^	68.30 ± 16.33 ^abc^	26.97 ± 5.80 ^b^	5.78 ± 1.58 ^d^	85.98 ± 28.83 ^ab^
AC + LC + MB	129.59 ± 6.48 ^b^	46.72 ± 12.06 ^b^	56.07 ± 2.77 ^bc^	23.68 ± 8.70 ^b^	5.70 ± 1.70 ^d^	44.07 ± 13.14 ^c^
CM	239.12 ± 25.34 ^a^	66.89 ± 17.03 ^ab^	45.45 ± 7.10 ^c^	56.81 ± 13.87 ^a^	14.08 ± 3.22 ^a^	58.66 ± 10.72 ^bc^
SEM	8.742	5.972	8.961	3.753	0.977	8.969
*p*-value	<0.001	0.024	0.016	<0.001	<0.001	0.021

IVDMD, in vitro dry matter digestibility; IVNDFD, in vitro neutral detergent fiber digestibility; IVADFD, in vitro acid detergent fiber digestibility; GP, gas production; DM, dry matter; MB, molasses + *Lc. casei* TH14; AC, Acremonium cellulase; LC, laccase; AC + LC, Acremonium cellulase + laccase; AC + LC + MB, Acremonium cellulase + laccase+ molasses + *Lc. casei* TH14; CM, chemical; SEM, standard error of the means. Data are presented as mean ± standard deviation. Values are means of four silage samples. ^a–d^ Means within columns with different superscripts differ at *p* < 0.05.

**Table 6:** Volatile fatty acid concentrations of rehydrated rice husk silages after 24 h of incubation with rumen inoculum.

Treatments	TVFA	Acetic Acid	Propionic Acid	Butyric Acid	Valeric Acid
	mmol/L	% of TVFA			
Control	48.62 ± 1.40 ^b^	46.19 ± 1.09	38.11 ± 2.16	14.19 ± 1.38 ^a^	1.51 ± 0.19 ^a^
MB	58.65 ± 3.12 ^a^	47.51 ± 0.53	36.98 ± 1.58	13.98 ± 0.96 ^a^	1.53 ± 0.12 ^a^
AC	49.89 ± 6.05 ^b^	47.77 ± 0.24	36.93 ± 2.49	13.77 ± 2.56 ^a^	1.53 ± 0.13 ^a^
LC	63.58 ± 1.89 ^a^	47.14 ± 1.26	38.26 ± 0.55	13.26 ± 0.91 ^ab^	1.35 ± 0.03 ^a^
AC + LC	49.52 ± 3.32 ^b^	45.89 ± 0.54	39.87 ± 0.96	12.85 ± 0.54 ^abc^	1.39 ± 0.03 ^a^
AC + LC + MB	47.49 ± 4.17 ^b^	47.78 ± 2.80	39.89 ± 2.96	10.97 ± 0.67 ^c^	1.37 ± 0.10 ^a^
CM	61.28 ± 3.62 ^a^	49.33 ± 1.35	38.88 ± 1.56	11.12 ± 0.39 ^bc^	0.68 ± 0.05 ^b^
SEM	1.844	0.752	1.003	0.614	0.053
*p*-value	<0.001	0.180	0.364	0.015	<0.001

TVFA, total volatile fatty acid; MB, molasses + *Lc. casei* TH14; AC, Acremonium cellulase; LC, laccase; AC + LC, Acremonium cellulase + laccase; AC + LC + MB, Acremonium cellulase + laccase+ molasses + *Lc. casei* TH14; CM, chemical; SEM, standard error of the means. Data are presented as mean ± standard deviation. Values are means of four silage samples. ^a–c^ Means within columns with different superscripts differ at *p* < 0.05.

## Data Availability

The original contributions presented in this study are included in the article. Further inquiries can be directed to the corresponding author.
